# Resistance to Rituximab Therapy and Local BAFF Overexpression in Sjögren’s Syndrome-Related Myoepithelial Sialadenitis and Low-Grade Parotid B-Cell Lymphoma

**DOI:** 10.2174/1874312900802010038

**Published:** 2008-05-28

**Authors:** Luca Quartuccio, Martina Fabris, Massimo Moretti, Francesca Barone, Michele Bombardieri, Maurizio Rupolo, Sandra Lombardi, Costantino Pitzalis, Carlo Alberto Beltrami, Francesco Curcio, Salvatore De Vita

**Affiliations:** 1 Clinic of Rheumatology, DPMSC, University of Udine, Italy; 2 Division of General Pathology, DPMSC, University of Udine, Udine, Italy; 3 Divisione di Reumatologia, Dipartimento di Clinica e Terapia Applicata, Università La Sapienza, Rome, Italy; 4 Centre for Experimental Medicine & Rheumatology, William Harvey Research Institute, St. Bartholomew's and Royal London School of Medicine, London, UK; 5 Department of Oncology, Centro di Riferimento Oncologico, IRCCS-National Cancer Institute, Aviano (PN), Italy; 6 Department of Pathology, DPMSC, University of Udine, Italy

## Abstract

**Objective:**

B-cell expansion is a key feature of Sjögren’s syndrome (SS). Accordingly, several studies have reported the benefits of B-cell depletion with anti-CD20 monoclonal antibody (Rituximab) in the treatment of glandular and extraglandular manifestations of SS. Patients with SS are at increased risk of lymphoma development. B-lymphocyte stimulator (BAFF) is an essential cytokine for the control of B-cell maturation and survival, and high levels of BAFF were described in the serum and salivary glands of SS patients, strongly suggesting a crucial role in the proliferation of B cells in SS.

**Patient and Methods:**

We describe the treatments employed, with particular regards to rituximab therapy, and the histopathologic and biologic studies, in particular BAFF levels in serum and in pathologic tissues before and after B-cell depletion therapy, and the characterization of the cultured epithelial cells obtained by the parotid gland MALT-lymphoma, in a case of a 51-year old woman with primary SS and mixed cryoglobulinaemia type II with features of systemic vasculitis, who developed a bilateral parotid MALT-type lymphoma. Rheumatoid factor (RF), cryoglobulins, BAFF levels were assessed monthly up to month +6, then at the end of follow-up (month +12), as well as peripheral blood CD19-positive B-cell level

**Results:**

A significant systemic effect of rituximab on B-cell biomarkers was documented, however, the cryoglobulinemic syndrome did not improve and the parotid enlargement did not decrease confirming the failure of B-cell depletion to affect the parotid lymphoma. BAFF levels decreased only under B-cell depletion associated with high-dose steroids. Tissue studies further documented the persistent overexpression of BAFF in the salivary gland pathologic tissue during the disease course.

**Conclusion:**

Tissue and systemic overexpression of BAFF may have contributed to resistance to rituximab therapy, in MALT lymphoproliferation associated with SS. Thus, alternative treatment strategies should be then considered, possibly including BAFF-targeted approaches.

## INTRODUCTION

B-cell expansion is a key feature of Sjögren’s syndrome (SS), in particular for systemic vasculitis manifestations, and for lymphoma development [1]. Accordingly, preliminary studies have reported the possible benefits of B-cell depletion with rituximab in the treatment of glandular and extraglandular manifestations of primary SS [2]. B-cell activating factor (BAFF), also known as BLyS, plays a critical role in the B-cell survival. BAFF is expressed by T and B cells infiltrating the salivary glands in SS, as well as by ductal epithelial cells [3]. Abnormal BAFF expression has been also demonstrated to be involved in different autoimmune diseases and malignant lymphoproliferative disorders [4, 5]. Therefore, BAFF overexpression in the salivary gland microenvironment may sustain the local proliferation of B cells in SS, and may then influence in some degree the response to rituximab treatment in this disease. Herein, we report clinico-pathologic and biological follow-up studies on a SS patient with lymphoma treated unsuccessfully with rituximab. These studies highlight the overexpression of BAFF from pre-malignant to malignant lymphoproliferation of mucosa-associated lymphoid tissue (MALT)-type lymphoma in SS supporting the hypothesis of a role of BAFF for resistance to rituximab in some SS cases with MALT lymphoproliferation.

## CASE REPORT

### History

We describe the case of a 51-year old woman with primary SS and mixed cryoglobulinaemia type II with features of systemic vasculitis, i.e., purpura, peripheral neuropathy, and skin ulcers, which developed a bilateral parotid MALT-type lymphoma.

In 1997 primary SS was diagnosed based on subjective and objective dry mouth and dry eye manifestations, positive anti-Ro/SSA and anti-La/SSB antibodies and minor salivary gland biopsy showing grade 4 on Chisholm and Mason score [6]. In July 2002, given the persistence of right parotid enlargement of recent onset, the patient underwent right parotid biopsy diagnosed histologically as non-malignant myoepithelial sialoadenitis (MESA) with lymphoproliferative lesions [7], with the presence of a diffuse infiltrate consisting of B cells expressing CD20+/CD5–/CD10– phenotype and formation of islands of myoepithelial proliferation, in the lack of centrocyte-like cells forming broad interconnecting strands between lymphoepithelial lesions and broad “halos” around the epithelial cell nests. On the same sample, molecular analysis of variable, diversity and joining (V-D-J) region rearrangements of immunoglobulin heavy chain (IgH) genes amplified by polymerase chain reaction (PCR) [7] revealed a B-cell monoclonal expansion in the pathologic tissue. In September 2003 the patient was referred to our Clinic because of persistent bilateral parotid gland swelling, purpura and paresthesias on the lower limbs, with a perimalleolar ulcer 2 x 3 cm on the right leg. Hepatitis B and C virus infections were absent. Electromyography demonstrated a mild sensitive axonal polyneuropathy on the lower limbs. Serum rheumatoid factor (RF), cryoglobulins and complement fraction C4 levels were 9190 IU/L (normal value <20 IU/L), 3256 mg/dl and 7 mg/dl (normal value 10-40 mg/dl), respectively. A second right parotid biopsy showed histological features in keeping with the diagnosis of MALT-type B cell non Hodgkin’s lymphoma (NHL). B-cell monoclonal expansion in the NHL was detected by repeat V-D-J IgH chain rearrangements by PCR. NHL staging was IE, on the basis of negative computed tomography thorax and abdomen scan and negative bone marrow biopsy. In November 2003 four weekly infusions of rituximab 375 mg/m^2^ were administered without any clinical improvement of the skin ulcer, peripheral neuropathy and bilateral parotid swelling. Then, the patient also failed to show clinical improvement after other therapies (oral daily and pulse cyclophosphamide, 9 g in total, then azathioprine for three months), while a third repeated right parotid biopsy documented the persistence of MALT-type lymphoma (July 2004). On February 2005 immunosuppressive therapy was discontinued and the patient underwent subtotal bilateral parotidectomy. Histopathologic analysis showed a B cell MALT-type lymphoma in both parotid glands. One month after bilateral parotidectomy, the skin ulcer decreased by two thirds in size, and concomitantly the RF titre and cryoglobulin level decreased, in the absence of any immunosuppressive treatment. Given the persistence of skin ulcer and purpura and a biologically active disease (RF 3390 IU/L, cryoglobulins 440 mg/dl, C4 6 mg/dl), the patient underwent repeated courses of plasma exchange from May to June 2005, achieving an almost complete ulcer healing by August 2005. However, the patient experienced a new episode of purpura in the lower limbs and recurrence of the perimalleolar skin ulcer one month later (end of September). Plasma exchange was started again with the same induction schedule used before, but the skin ulcer worsened. In February 2006, right parotid swelling was noticed again. A fourth parotid biopsy (February 2006) showed a persistence of MALT-type lymphoma, and CT scan confirmed the bilateral enlargement of the parotid glands, consistent with local tumor relapse. Eight consecutive rituximab infusions (375 mg/m^2^ weekly), in association with high-dose steroids (1 mg/kg for 1 month, then slowly reduced to 5 mg of prednisone equivalents during the additional 4 months) were administered (Fig. **1**). RF, cryoglobulins, BAFF levels (measured by immunosorbent assay, R&amp;D Systems Quantikine ELISA kit) (Fig. **1**) were assessed monthly up to month +6, then at the end of follow-up (month +12), in conjunction with peripheral blood CD19-positive B-cell count, immunoglobulins, C3 and C4 (data not shown). This treatment regimen was well tolerated. However, despite a significant systemic effect of rituximab on B-cell biomarkers (reduction in RF, cryoglobulin and immunoglobulin levels, and persistent depletion in the peripheral blood CD19-positive B cells until month +6) the neuropathy did not improve, the skin ulcer was reduced only by 25% at month +6 and it worsened at month +12, and the parotid enlargement did not decrease, as demonstrated by CT scan and ultrasonography. Thus, prolonged rituximab therapy plus high-dose steroids did not prove effective in the lymphoproliferative and vasculitic disease.

### Detection of BAFF Serum Levels Before and After Rituximab

BAFF levels initially decreased but then increased again during steroid tapering (at doses <0.5 mg/kg/day of prednisone equivalents), remaining at high levels during the whole study period (Fig. **2**).

### BAFF Detection in the Pathologic Target Tissues

Tissue studies further documented the persistent overexpression of BAFF in the salivary gland pathologic tissue during the disease course. Immunohistological analysis of the parotid biopsy taken in July 2002 (parotid MESA) demonstrated strong local BAFF production. BAFF-positive cells were detected within the lymphocytic infiltrate, in particular in association with germinal centre-like structures as well as in ductal epithelium, and BAFF receptor (BAFF-R) was detected on the majority of the infiltrating lymphocytes and in particular in the B lymphocytes populating the germinal centre mantle zone (Fig. **2A**,**2C**).

In the lymphomatous parotid biopsy obtained in February 2005, after rituximab monotherapy, BAFF-positive cells were again detected within the dense lymphocytic infiltrate, both in the reactive areas and in the areas infiltrated by small monocytoid-like malignant B cells. BAFF-R expression was widely detected in the B cell area. BAFF and BAFF-R staining of the ductal epithelium was again detected as well (Fig. **2B**,**2D**).

### BAFF Expression in Salivary Gland Culture Studies

**Fig. (3). BAFF expression and secretion modulation by IFN-g stimulation on cultured epithelial cells. (A):**
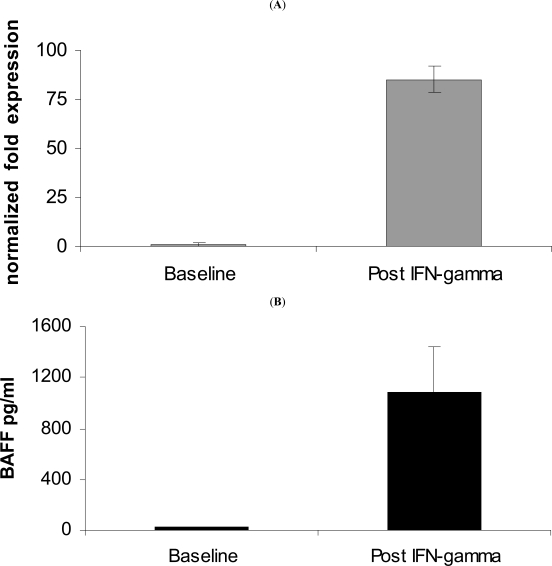
BAFF mRNA expression was increased more than 80 fold by stimulation with IFN-g 10 ug/ml for 48 hours (Real-time PCR, primers and PCR condition as previously described, ref. 10). (**B**) BAFF concentration in the supernatant increased about 50 fold (from 20110.1 pg/ml to 10811357 pg/ml; measured by immunosorbent assay, R&D Systems Quantikine ELISA kit). The experiments were done two times in duplicate.

The salivary gland epithelial cells (SGEC) obtained from the parotidectomy surgical specimen in February 2006 were maintained in culture (F12 Coon’s modified with 5% FBS, and bovine extracts) and characterized by the expression of epithelial markers, as previously described [8]. SGECs showed significant expression of BAFF both at the mRNA (semi-quantitative PCRs, data not shown) and at the protein levels (data not shown) [9]. Incubation of SGECs with IFN-γ (10 ng/ml, 48 hours) demonstrated a strong induction of BAFF mRNA expression (real-time PCR) and protein release in the supernatant (Fig. **3**) [10].

## DISCUSSION

Low-grade B-cell marginal zone MALT-type lymphoma, usually involving the parotid glands, is an important complication of primary SS [1]. Parotid lymphoma may evolve from parotis MESA, which may in turn present with different pathologic and molecular patterns of B cell proliferation [7]. The possible efficacy of rituximab monotherapy has been reported in parotid gland MALT-type lymphoma in primary SS, however with contrasting results [2, 11-18], also in our early experience [19]. Recently, Seror *et al*. [18] detected efficacy, however with different degrees of response, in 80% of MALT-type lymphoma in SS (Table **1**). Thus, non responders or partial responders to rituximab are also present in SS. The present paper is focused on the possible role of local BAFF in influencing the response to rituximab of parotid lymphoproliferative disorders of SS.

This is the first report where salivary glands BAFF expression has been assessed at different stages of progression towards parotid B-cell NHL in SS. BAFF expression within the salivary glands was found consistently elevated during the disease course, both before and after rituximab treatment. BAFF expression was detected in the infiltrating inflammatory cells confirming previously published results [3]. Moreover, diffuse BAFF expression was detected on the ductal epithelium also in parotid MESA and lymphoma SS-related, and BAFF expression and protein synthesis were confirmed by the study of SGECs established from the MALT lymphoma of the patient. This further highlights the role of the epithelial cells in SS pathogenesis. These data extend previous results in epithelial cells from benign minor salivary gland lesions in SS [10]. The epithelial salivary cells appear then involved not only in the process of antigen presentation, but also in the survival of the infiltrating B cells in SS. A possible BAFF autocrine loop may occur also in the epithelial compartment in SS, besides the autocrine loop hypothesized in proliferating B-cells [9].

After repeated and more prolonged rituximab therapy combined with high-dose corticosteroids serum BAFF levels somewhat decreased from baseline (but did not normalize) when the steroid dose was maintained between 1 mg/kg/day and 0.5 mg/kg/day. Then BAFF rapidly increased again when the steroid dose was reduced below 0,5 mg/kg/day (Fig. **2**). Serum RF and cryoglobulins decreased and then increased accordingly (Fig. **1**). The present report then confirms previous data about the partial downregulation of BAFF production under medium to high doses of steroids [20], which were used with this purpose. However, such a partial decrease in BAFF may be clinically ineffective, and rituximab may increase BAFF and then in part inhibit such steroid-induced BAFF decrease [21]. Overall, as revealed by the bilateral parotid swelling, confirmed by CT scan imaging and ultrasonography, there was a failure of the combination therapy of steroids and rituximab as used in our case. On the other hand, a more prolonged high-dose steroid therapy, possibly keeping a more prolonged BAFF suppression, is not advisable in our opinion, due to the possible severe side effects. Overall, after literature revision and including the present case in all the SS patients with MALT-type lymphoma treated with rituximab not in the context of chemotherapeutic regimen or in association with local radiotherapy, rituximab treatment seems to be less effective. When analyzing the subset of MALT-type lymphoma involving the salivary glands (Table **1**), some response is seen in less than half of the patients [2, 11-18]. The response to rituximab of SS-associated lymphoma may be also different with regard to the different histotype of lymphoma and to the tissue involved [22]. Rituximab alone may fail to deplete B-cells in the target tissue, due to the resistance of peculiar B-cell subtypes, e.g., MZ B cells, and to microenvironment local factors, as recently well described in a murine model [23]. In these mice, tissue BAFF expression reduced the effect of B-cell depletion, while concomitant BAFF inhibition allowed to reach much higher degrees of B-cell depletion [23].

In conclusion, clinico-pathologic and biological follow-up studies allowed to highlight overexpression of BAFF from pre-malignant to malignant B-cell lymphoproliferation of MALT in SS. Lymphoproliferation was resistant to rituximab therapy, even when associated with high dose corticosteroids. Targeting directly the B-cell compartment may be then promising for some SS clinical features, but other novel therapeutic strategies, targeting indirectly the B cells, e.g., anti-BAFF agents, should be explored, with possible drug combination [24].

## Figures and Tables

**Fig. (1). Serological changes of rheumatoid factor (RF), cryoglobulins (cryos), BLyS levels and dose changes of daily methylprednisolone (MP) during rituximab (RTX) 650 mg weekly for 8 weeks) infusions and in the subsequent follow-up.**
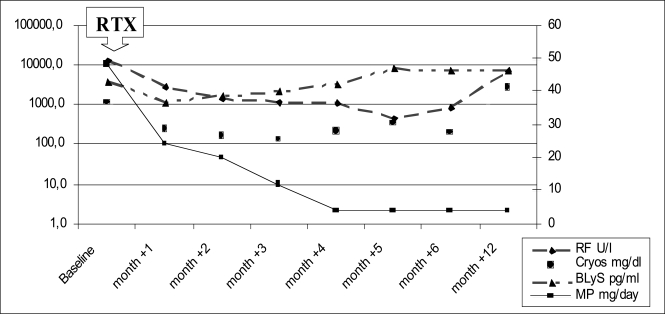
RF, cryos and BLyS levels are expressed in logarithmic scale on the left side. The scale on the right side of the graphic is related to MP daily dose

**Fig. (2). BAFF and BAFF-R expression in parotid gland (MESA pre-rituximab in the Panel A and C, and MALT NHL postrituximab in the Panel B and D) during the follow-up.**
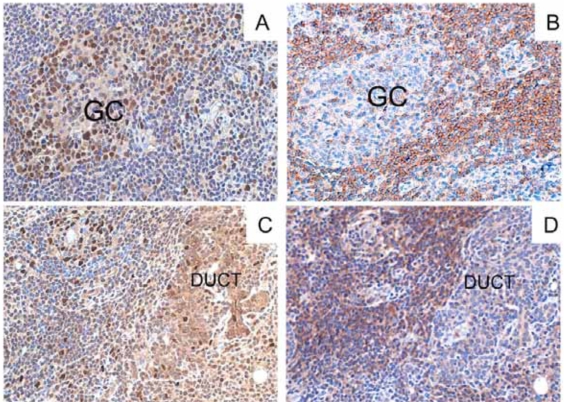
Microphotograph showing diffuse BAFF (**A** and **B**) and BAFF-R (**C** and **D**) expression in the salivary gland of the patient in the parotid biopsies relative to MESA 2002 (**A** and **C**) and post-rituximab MALT NHL 2005 (**B** and **D**). BAFF strongly positive cells (brown) were detected in the MESA both inside the infiltrated areas (germinal centres=GC in panel **A**) and in the ducts (data not shown), but intense BAFF expression with the same pattern were detected also in the B-cell MALT NHL despite the treatment with rituximab (panel **B**). Sequential sections show strong expression of BAFF-R in mantle zone B cells (panel **C** for the MESA), and in the infiltrating lymphocytes surrounding the epithelial structures (panel **D** for the MALT NHL) (Original magnification 100x).

**Table 1:** Cases With Primary SS and Lymphoma Treated with Rituximab

*Author, Years*	*N. of Pts*	*Type of Lymphoma/Bone Marrow Involvement*	*Ann Arbor Stage*	*Response*	*Other Concomitantly Immunosuppressive Agents*
Somer, 2003	1	Parotid gland MALT-type/no	IE	Yes	No
Voulgarelis, 2004*	4	Salivary gland MALT-type/yes* Nodal marginal zone/no* Pulmonary MALT-type/yes* Salivary gland MALT-type/yes*	IV IIE IV IV	Yes Yes Yes Yes	CHOP CHOP CHOP CHOP
Harner, 2004	1	Nodal marginal zone/Pulmonary MALT-type/no	IIE	Yes	No
Ramos-Casals, 2004	2	Ovarian MALT-type/yes Ocular MALT-type/yes	IV IV	Yes Yes	CHOP Local radiotherapy
Pijpe, 2005	1	Parotid gland MALT-type/no	IE	Yes	No
Gottenberg, 2005#	2	Digestive tract MALT-type/no# Salivary gland MALT-type/no#	IE IE	Yes No	MP 500 mg x 4, HQ No
Pijpe, 2005	7	Parotid gland MALT-type/no Parotid gland MALT-type/no Parotid gland MALT-type/no Parotid gland MALT-type/no Parotid gland MALT-type/no Parotid gland MALT-type/no	IE IE IE IE IE IE IE	No No Yes Yes No Yes No	No PDN 15 mg/day No No PDN 7.5 mg/day, MTX PDN 5 mg/day, AZA No
Voulgarelis, 2006*	6	Nodal marginal zone/no DLBCL/no Salivary gland MALT-type/yes *Nodal marginal zone/no* Pulmonary MALT-type/yes* Salivary gland MALT-type/yes*	II II IV IIE IV IV	Yes Yes Yes Yes Yes Yes	CHOP CHOP CHOP CHOP CHOP CHOP
Seror, 2007#	5	Salivary gland MALT-type/no# Nodal marginal zone/yes Gastric/pulmonary MALT-type/no Gastric MALT-type/no# DLBCL/no	IE IV IV IE III	No Yes Yes Yes Yes	No No Mini-CHOP HQ CHOP
Present report	1	Parotid gland MALT-type/no	IE	No	High-dose steroids

* There is some patients^’^ overlapping between the cases reported in Voulgarelis, 2004 and Voulgarelis, 2006.

# There is some patients^’^ overlapping between the cases reported in Gottenberg, 2005 and Seror, 2007.

Abbreviations: pts, patients; MALT, mucosa-associated lymphoid tissue; CHOP, cyclophosphamide, doxorubicin, vincristine, prednisone; AZA, azathioprine; PDN, prednisone;MTX, methotrexate; MP, methylprednisolone; DLBCL, diffuse large B-cell lymphoma; HQ, hydroxychloroquine.
